# Reliability of Objectively Measured Sedentary Time and Physical Activity in Adults

**DOI:** 10.1371/journal.pone.0133296

**Published:** 2015-07-20

**Authors:** Eivind Aadland, Einar Ylvisåker

**Affiliations:** Faculty of Teacher Education and Sport, Sogn og Fjordane University College, Sogndal, Norway; Universidad Pablo de Olavide, Centro Andaluz de Biología del Desarrollo-CSIC, SPAIN

## Abstract

**Background:**

In adults, a minimum of 3–5 days of accelerometer monitoring is usually considered appropriate to obtain reliable estimates of physical activity (PA). However, a longer period of measurement might be needed to obtain reliable estimates of sedentary behavior (SED). The aim of this study was to determine the reliability of objectively assessed SED and PA in adults.

**Methods:**

Eighty-seven adult subjects (28 men; mean (standard deviation) age 31.3 (12.2) years; body mass index 23.7 (3.1) kg/m^2^) wore the GT3X+ accelerometer for 21 subsequent days, for which the reliability of different wear time criteria (8 to 12 h/day and 3 to 5 d/week) was explored. Variance partitioning along with the Spearman-Brown prophecy formula was used as the basis for determining intraclass-correlation coefficients (ICC) and the number of monitoring days needed (N) to achieve an ICC = 0.80. Week-by-week reliability was reported using ICC, Bland-Altman plots and absolute measures of agreement.

**Results:**

Seven-10 days of monitoring was needed to reliably assess overall- (axis 1 and vector magnitude (VM) counts per minute (CPM)) and moderate-to-vigorous PA (MVPA), 3–4 days was needed for light PA (LPA), whereas the number of days needed for SED depended on whether adjustments were made for wear time (6–8 days) or not (13–15 days). The week-by-week ICC was ≥0.70 for all variables, with limits of agreement being ±267.8 cpm for CPM, ±352.3 cpm for VM CPM, ±76.8 min/day for SED, ±57.8 min/day for LPA and ±43.8 min/day for MVPA, equal to 1.0–1.6 standard deviations, when adjustment was made for wear time.

**Conclusions:**

For most variables, more than one week of measurement was needed to achieve an ICC = 0.80. Correcting for wear time was crucial to reliably determine SED. Considerable week-by-week variability was found for all variables. Researchers need to be aware of substantial intra-individual variability in accelerometer-measurements.

## Introduction

Accelerometry has gained widespread popularity as a valid tool for the assessment of free-living physical activity (PA) and sedentary behavior (SED) in diverse study settings. Yet, given the inherent variation in behavior over time, an important aspect of accelerometer measurements is how many days of measurement to be considered to obtain reliable results. Importantly, whether PA is measured as a predictor (x-) variable or an outcome (y-) variable, noise will hamper researchers’ ability to make valid causal inferences in PA epidemiology [1] and possibly misinform the society regarding targets for public health management.

In adults, ≥3–5 days of monitoring are normally considered appropriate, which is in accordance with recommendations given [2]. However, estimates of how many days of monitoring that should be included to obtain a reliable result vary considerably between studies [3–7, 2, 8], and might also vary between outcome variables of interest [6, 8]. According to Matthews et al [6], inclusion of more days may be needed to arrive at reliable estimates (intraclass correlation coefficient (ICC) ≥0.80) for “physical inactivity” (<500 cpm from the Actigraph 7164) (≥7 days), compared to PA (≥ 3–4 days). A comparable finding has been shown in older adults, where 2–3 days was needed for PA, whereas 5 days of monitoring was needed for SED (<50 cpm from the Actigraph 7164). The possible impaired reliability for SED compared to other variables may be of critical importance, given the increased interest in SED in the primary and secondary prevention of a range of chronic diseases as well as premature death [9–11]. Inconsistent conclusions across studies might amongst other reasons arrive from unreliable measurements of SED, as most of these studies have included ≥3–5 days of measurement [12–17], with some exceptions (≥1 day [18]; ≥6–7 days [19, 20]). Moreover, as SED are likely to be related to wear time, correction for wear time might improve reliability. Consistent with this hypothesis, percent SED has previously been shown to be superior to minutes of SED as a predictor of metabolic risk [18].

Historically, sedentary behavior (SED) was conceptualized as the lower end of the PA spectrum, as opposed to moderate- to vigorous PA (MVPA), but is now increasingly being viewed as a behavior distinct from PA, defined as waking behavior characterized by an energy expenditure ≤ 1.5 metabolic equivalents (METs) [21]. Although somewhat arbitrary, studies using Actigraph instruments mainly operationalize SED as the time spent <100 cpm. This cut-off differs substantially from that used to define “inactivity” (<500 cpm) applied by Matthews et al [6]. Thus, besides the study of reliability of SED in older adults [8], to the best of our knowledge, reliability of SED obtained by accelerometry have not been investigated in adults, and should be prioritized [22]. Furthermore, no studies have determined the intra-individual week-by-week agreement of accelerometer outcomes using absolute measures of reliability (i.e., standard error of the measurement and limits of agreement). Most evidence suggest that a reliability of ≥0.70–0.80 are achieved for PA with 3–7 days of monitoring by estimation of the number of days needed based on the Spearman Brown prophecy formula, when measurements are conducted over a single 7-day period [5–7, 2, 8]. However, such study designs have received critique for possibly leaving to optimistic results and should be interpreted with caution [23–25]. First, the results are in principle only generalizable to the included days, as inclusion of additional days, weeks or seasons will add variability. Secondly, the assumption of compound symmetry (i.e., similar variances and co-variances) across days of measurement might not be fulfilled. Additionally, ICC is the variance partitioning of subjects to the total variance, thus ICC is a relative and context-specific estimate that depends on the heterogeneity of the sample [26–28]. Thus, research targeting agreement of SED and PA measurements by means of absolute measures of reliability, which allow for a direct quantification of how much outcomes vary over time independent of the variability of observations should be given priority.

The aim of the present study was to determine the agreement of objectively assessed SED and PA over 3 subsequent weeks in an adult population. Based on previous findings [8, 6], we hypothesized that a longer monitoring period would be needed to obtain reliable estimates of SED, compared to other outcomes.

## Material and Methods

### Subjects

Eighty-seven subjects were recruited by word of mouth mainly among sport science students and staff at the Sogn og Fjordane University College, Norway for a long-term (21 days) objective measurement of PA level during the spring 2014. Inclusion criterion were an age of 18 years or older. No specific exclusion criteria were applied, besides being unable to move normally (e.g. being severely injured, using crutches, wheelchair etc.) during the period of monitoring. Potential subjects received oral and written information about the study, which had no foreseeable harms. As the study was conducted anonymously, the subjects provided their consent to participate by actively choosing to wear the accelerometer. The study is reviewed by the Regional Committee for Medical and Health Research Ethics of Western Norway.

### Procedures

Physical activity was measured using the Actigraph GT3X+ accelerometer (firmware 2.2.1) (Pensacola, FL, USA). Two accelerometers were attached to an elastic belt, and worn at contralateral hips over a period of 21 days. Half-way through the measurement period (day 11), subjects switched the accelerometers units around (1 unit was worn on the right side the first 10 days and at the left side the last 10 days, and vice versa), two allow for a within-accelerometer controlled study off contralateral hip differences. As differences between the hips were absolutely minimal (effect size ≤0.04), all results are based on output from one of the units for the purpose of the present study. Subjects were instructed to wear the accelerometer at all times, except during water activities (swimming, showering) or while sleeping. Units were initialized at a sampling rate of 30 Hz. Files were analyzed at 10 second epochs using the Kinesoft v. 3.3.75 software [29]. Data was analyzed using different criteria for valid wear time (≥8; ≥10; ≥12 hours/day). In all analyses, consecutive periods of ≥60 minutes of zero counts (allowing for ≤2 minutes of non-zero counts) were defined as non-wear time [2, 30] and excluded prior to scoring.

Results were initially reported for overall PA level (CPM), as well as SED (<100 cpm), light PA (LPA) (100–2019 cpm), MPA (2020–5998 cpm), VPA (≥5999 cpm) and MVPA (≥2020 cpm) obtained from the vertical axis (axis 1) [31], as well as the vector magnitude (VM) CPM. However, as the level of VPA were very low (median (interquartile range) 1.3 (9.0) min/day), MPA and VPA were collapsed and only MVPA were reported throughout the study. Intensity-specific PA and SED were reported as minutes/day and as percentage values of daily valid wear time.

Subjects characteristics (sex, age, body mass and height) were self-reported. Body mass index (BMI) was calculated as the body mass divided by the squared height (kg/m^2^).

### Statistical analyses

Subject characteristics were reported as means and standard deviations (SD).

The association between wear time and PA and SED were investigated using linear mixed model regression analyses including a random intercept for subjects. Differences between week- and weekend days were tested by including a dummy variable (week- vs. weekend day) in the above-mentioned model (i.e., adjustment were made for wear time). Residuals were normally distributed in all models. Findings were reported as regression coefficients and 95% confidence intervals (CI). Further, we used variance partitioning obtained from a two-way mixed effect model reporting the ratio of week-weekend variance to the total variance (week-weekend variance/(between subject variance + week-weekend variance + residual variance)), including wear time as a fixed effect [32]. A wear time criterion of ≥10 hours/day was used for these analyses.

The single-day reliability and number of days needed to obtain the desired reliability was assessed for wear times of ≥8; ≥10; ≥12 hours/day. Reliability for single days of measurement was assessed using variance partitioning obtained through a one-way random effect model, reporting the ratio of the between subject variance to the total variance (between subject variance/(between subject variance + residual variance)) [26]. Number of days needed to obtain a reliability of 0.80 was estimated using the Spearman Brown prophecy formula [2, 32]: N = ICC_t_/(1-ICC_t_)*[(1-ICC_s_)/ICC_s_], where N = number of days needed, ICC_t_ = desired level of reliability, and ICC_s_ = reliability for single days.

Bland Altman plots, showing the difference between two subsequent weeks as a function of the mean of the two weeks [26], was applied to determine the week-by-week variability. Because the data generally were deemed to be homoscedastic (y not related to x), the standard error of the measurement (SEM) and limits of agreement (LoA) were calculated from the residual variance (i.e., within-subjects) error term obtained through a one-way random effect model using the 3 accumulated weekly means (as opposed to 21 single day observations) as the dependent variable (SEM = √residual variance; LoA = SEM*√2*1.96) [28]. As the pattern of variability for overall PA and MVPA were heteroscedastic (y related to x), we also reported the percentage typical error (SEM/median values*100), which are more appropriate when variability depends on the value of the measurement. Finally, results were also reported with adjustment for wear time by including wear time as a fixed effect in a two-way mixed effect model [32].

All analyses were performed using IBM SPSS v. 20 (IBM SPSS Statistics for Windows, Armonk, NY: IBM Corp., USA). A p-value < .05 indicated statistically significant findings.

## Results

### Subject characteristics

All 87 subjects (28 (32%) men and 59 women; age 31.3 (12.2) years; body mass 70.4 (12.2) kg, height 172.1 (8.1) cm, body mass index 23.7 (3.1) kg/m^2^) was included in the analyses. Weekly mean (SD) SED and PA across the three weeks were: CPM = 486 (172); VM CPM = 878 (242); SED = 564 (63) min/day / 69.5 (6.2)%; LPA = 183 (57) min/day / 22.3 (5.8)%; MVPA = 65.9 (28.1) min/day / 8.2 (3.6)%.

### Association between wear time and physical activity

Wear time was positively associated with absolute time in SED, LPA and MVPA, but was fully attenuated for LPA and SED when reported as percentage values (Table 1). On the contrary, wear time was negatively related to CPM and percentage time in MVPA, whereas no relationship was found for VM CPM.

**Table 1:** Association between wear time and physical activity.

	β (95% CI)	F (*p*)
**CPM**	-0.190 (-0.316–-0.062)	8.5 (.004)
**VM CPM**	-0.033 (-0.206–0.140)	0.1 (.709)
**SED**		
Min/day	0.709 (0.676–0.743)	1704 (< .001)
%	1.59E-3 (-2.60E-3–5.79E-3)	0.6 (.457)
**LPA**		
Min/day	0.238 (0.212–0.263)	338 (< .001)
%	1.55E-3 (-1.57E-3–4.67E-3)	1.0 (.330)
**MVPA**		
Min/day	0.047 (0.026–0.067)	19.8 (< .001)
%	-3.94E-3 (-6.52E-3–-1.35E-3)	8.9 (.003)

The association between wear time and the different outcome variables for physical activity and sedentary behavior.

CPM = counts per minute; VM CPM = vector magnitude counts per minute; SED = sedentary time; LPA = light physical activity; MVPA = moderate-to-vigorous physical activity; CI = confidence interval

### Reliability for ≥8 to ≥12 h criteria to define a valid day

The number of days that were available for analysis declined as a result of applying a more strict wear time criteria (n = 1423 (78%), n = 1222 (67%) and n = 947 (52%) for ≥8, ≥10 and ≥12 hours/day, respectively). Table 2 shows the reliability for single days of measurement (ICC_s_) and number of days (N) needed to achieve a reliability of 0.80. Reliability increased with a stricter wear time criteria. For overall PA level, 7 to 10 (CPM) and 6 to 9 (VM CPM) days was needed to achieve the desired reliability. The best reliability was found for LPA, where 3 to 4 days was needed across all wear time criteria. For MVPA, 7 to 10 days was needed. Number of days needed for SED, differed substantially according to whether SED was reported as an absolute value (13 to 16 days) or as a percentage value of wear time (6 to 8 days).

**Table 2:** Estimated number of days needed to achieve a reliability of 0.80.

	Wear time criteria for a valid day					
	≥8 hours		≥10 hours		≥12 hours	
	ICC_s_	N	ICC_s_	N	ICC_s_	N
**CPM**	0.29	9.8	0.31	8.9	0.35	7.5
**VM CPM**	0.31	9.0	0.34	7.8	0.38	6.6
**SED**						
Min/day	0.20	15.7	0.21	15.1	0.23	13.4
%	0.33	8.3	0.35	7.3	0.39	6.4
**LPA**						
Min/day	0.53	3.6	0.53	3.6	0.52	3.6
%	0.47	4.4	0.50	4.0	0.52	3.7
**MVPA**						
Min/day	0.29	9.7	0.31	8.7	0.35	7.4
%	0.32	8.5	0.34	7.8	0.36	7.0

Reliability for single days of measurement (ICC_s_) and number of days needed to achieve a reliability of 0.80 (N) for different criteria to determine a valid day of measurement.

CPM = counts per minute; VM CPM = vector magnitude counts per minute; SED = sedentary time; LPA = light physical activity; MVPA = moderate-to-vigorous physical activity; ICC_s_ = ICC for a single day of measurement; N = number of days needed to achieve a ICC = 0.80

Physical activity level were higher (β (95% CI) for CPM: 78.4 (49.8–107.0); VM CPM: 110.1 (71.5–148.8); LPA: 7.1 (1.4–12.7) min/day and 0.7 (0.0–1.4)%; MVPA: 11.3 (6.7–16.0) min/day and 1.5 (0.9–2.1)%) and SED were lower (-18.2 (-25.7–-10.6) min/day and -2.2 (-3.2–-1.3)%) on week days (n = 919) compared to weekend days (n = 303). Still, the week-weekend difference explained less than 5% of the total variation among all variables analyzed (ICC = 0.004–0.043).

### Reliability for three consecutive weeks of measurement

**Fig 1:**
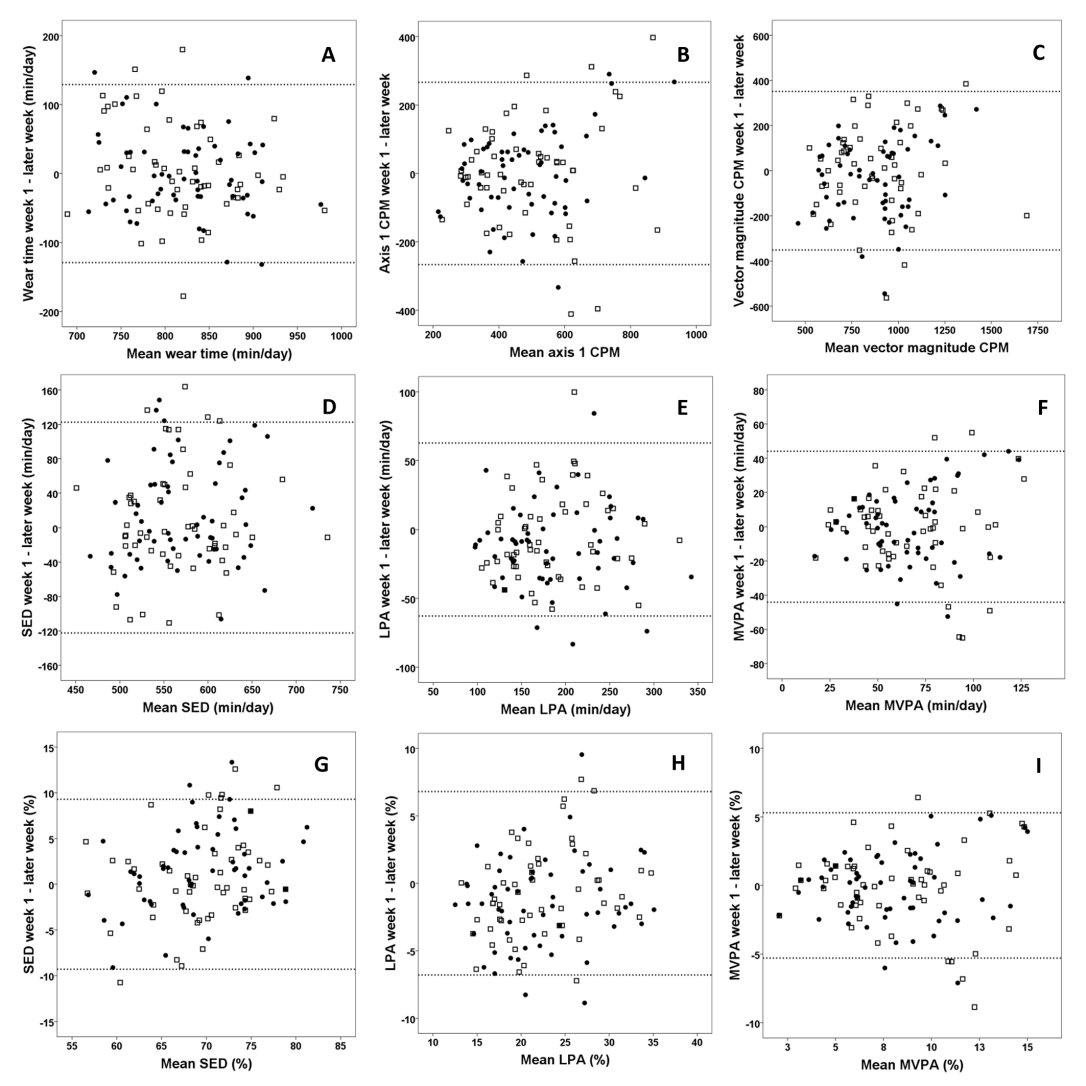
Bland Altman plots of measurement variability for different outcome variables. Bland Altman plots (the difference between two weeks of measurement on the y-axis versus the mean of the two weeks on the x-axis) for wear time (a); average counts per minute (CPM) from the vertical axis (b) and the vector magnitude (VM CPM) (c); absolute time spent sedentary (SED) (d), light physical activity (LPA) (e) and moderate-to-vigorous physical activity (MVPA) (f); percentage time in SED (g), LPA (h) and MVPA (i). Results are based on a ≥10 hours & ≥4 days wear time criteria. Filled dots are week 1–week 2 (n = 59); open squares are week 1–week 3 (n = 53)95% limits of agreement are indicated as dotted lines.

Table 3 and Fig 1 shows the week-by-week reliability using a ≥10 hours & ≥4 days wear time criteria. In total, 192 (74%) weeks with a wear time of 813 (71) min/day over 4 (20.3%), 5 (18.2%), 6 (29.2%) and 7 (32.3%) days of measurement were included in the analyses. An ICC ≥0.70 was achieved for all variables, except for SED (min/day). However, when adjusted for wear time, results for SED (min/day) improved markedly and in terms of ICC became identical to SED (%). For SED and LPA, the variability between weeks decreased when adjustment was made for wear time (-37 and -8%, respectively). This finding is consistent with a substantial variation in wear time across weeks (SEM (%) / LoA = 46.6 (5.7) / 129.1 min/day), given the relationships with LPA and SED as shown previously (Table 1). Other variables were more or less unaffected by the adjustment for wear time. Although nearly all variables reached an ICC ≥0.70, the absolute measures of reliability clearly shows that a substantial degree of individual variability must be expected across subsequent weeks. After adjustment for wear time, LoAs was 1.0–1.6 times the sample SDs. When typical errors were reported as a percentage value of the mean, the greatest variability vas found for MVPA (~24%) and the lowest for SED (~5%).

**Table 3:** Week-by-week reliability and the influence of wear time.

	Crude^1^			Adjusted for wear time^2^		
	ICC_s_	SEM (%)	LoA	ICC_s_	SEM (%)	LoA
**CPM**	0.71	96.1 (19.8)	266.4	0.70	96.6 (19.9)	267.8
**VM CPM**	0.75	126.7 (14.4)	351.2	0.75	127.1 (14.5)	352.3
**SED**						
Min/day	0.52	44.2 (7.8)	122.4	0.71	27.7 (4.9)	76.8
%	0.72	3.4 (4.9)	9.3	0.71	3.4 (4.9)	9.3
**LPA**						
Min/day	0.84	22.7 (12.4)	62.8	0.80	20.8 (11.4)	57.8
%	0.82	2.5 (11.2)	6.8	0.80	2.5 (11.2)	7.0
**MVPA**						
Min/day	0.70	15.9 (24.1)	44.1	0.71	15.8 (24.0)	43.8
%	0.75	1.9 (23.2)	5.3	0.73	1.9 (23.2)	5.3

The week-by-week reliability for different outcome variables for three consecutive weeks of measurement without- and with adjustment for wear time, using wear time criteria of ≥10 hours/day and ≥4 valid days.

CPM = counts per minute; VM CPM = vector magnitude counts per minute; SED = sedentary time; LPA = light physical activity; MVPA = moderate-to-vigorous physical activity; ICC_s_ = ICC for a single day of measurement; SEM (%) = standard error of the measurement (percentage of the mean value); LoA = limits of agreement

^1^Analyzed by means of a one-way random effect model

^2^Analyzed by means of a two-way mixed effect model.

We found slight improvements in week-by-week reliability when data was accumulated over longer (8 to 12 h) and more days (3 to 5 d), however, the pattern was not very consistent and the differences were minor (Table 4).

**Table 4:** Week-by-week reliability according to different wear criteria.

	Wear time criteria								
	≥8 hours/day			≥10 hours/day			≥12 hours/day		
	≥3 days	≥4 days	≥5 days	≥3 days	≥4 days	≥5 days	≥3 days	≥4 days	≥5 days
**N (%) weeks**	**234 (90)**	**219 (84)**	**204 (78)**	**217 (83)**	**192 (74)**	**153 (59)**	**202 (77)**	**168 (64)**	**135 (52)**
**CPM**									
ICC_s_	0.68	0.68	0.69	0.68	0.71	0.67	0.65	0.73	0.71
SEM	101.1	100.1	98.3	101.5	96.1	91.1	108.1	91.0	86.5
**VM CPM**									
ICC_s_	0.70	0.72	0.69	0.72	0.75	0.71	0.70	0.75	0.74
SEM	135.1	128.9	128.9	135.8	126.7	121.7	144.5	125.9	116.1
**SED (min/day)**									
ICC_s_	0.49	0.56	0.58	0.51	0.52	0.46	0.42	0.46	0.50
SEM	48.8	45.4	44.5	43.9	44.2	43.7	50.3	48.9	43.2
**SED (%)**									
ICC_s_	0.70	0.72	0.73	0.71	0.72	0.75	0.69	0.71	0.75
SEM	3.3	3.2	3.1	3.4	3.4	3.1	3.6	3.4	3.1
**LPA (min/day)**									
ICC_s_	0.83	0.84	0.84	0.84	0.84	0.86	0.83	0.84	0.86
SEM	23.6	23.4	23.1	23.0	22.7	20.8	23.2	22.2	20.6
**LPA (%)**									
ICC_s_	0.80	0.82	0.83	0.81	0.82	0.85	0.78	0.81	0.84
SEM	2.6	2.5	2.4	2.6	2.5	2.3	2.7	2.5	2.3
**MVPA (min/day)**									
ICC_s_	0.70	0.69	0.69	0.68	0.70	0.68	0.67	0.71	0.68
SEM	15.0	15.1	15.2	16.6	15.9	15.2	17.6	15.7	15.5
**MVPA (%)**									
ICC_s_	0.71	0.71	0.72	0.73	0.75	0.70	0.72	0.75	0.71
SEM	2.0	2.0	2.0	2.0	1.9	1.8	2.1	1.9	1.8

The week-by-week reliability for different outcome variables for different criteria to determine a valid day- and week of measurement.

CPM = counts per minute; VM CPM = vector magnitude counts per minute; SED = sedentary time; LPA = light physical activity; MVPA = moderate-to-vigorous physical activity; ICC_s_ = ICC for a single day of measurement; SEM = standard error of the measurement; Limits of agreement can be calculated as SEM*√2*1.96

## Discussion

The present study is the first to investigate the week-by-week reliability of measurements of SED and PA, as obtained by accelerometry in adults, using absolute measures of reliability. There was a clear difference in reliability for SED reported in terms of minutes/day (absolute measure) compared to as a percentage value of the valid wear time (relative measure), favoring the latter. This disparate finding is caused by the strong relationship between wear time and SED. Thus, adjustment for wear time reduced the variability in SED (min/day) considerably and left similar ICC as for SED (%). Absolute measures of reliability showed that week-by-week differences must be expected to be substantial for all outcome variables. Contrary to our hypothesis, the reliability of SED and PA were similar, or, when variability was reported relative to the mean values; considerably better for SED compared to PA.

Although wear time criteria differ between studies, many studies employ a wear time criteria of ≥10 hours/day for ≥3–5 days/week to constitute a valid accelerometer measurement in adults [22, 16, 14, 17, 13, 12]. These criteria are in line with current recommendations [22, 2]. The present findings suggest that the number of days to be considered a valid measurement might be increased to a full week or longer to achieve an ICC of 0.80, which is currently the most applied desired reliability level. Consistent with previous studies [6, 8], we found impaired reliability for SED (min/day) compared to PA. This could indicate that SED is a behavior that are difficult to capture as it might vary more on a day-by-day basis than other activities that are performed on a higher intensity-level. However, SED given as a relative measure (percentage of valid wear time) was superior to SED reported in absolute term (minutes/day) (requiring 7 vs. 15 days of measurement for the ≥10 h criteria) in the present study. This finding results from a considerable day-to-day variation in wear time, given the very strong relationship between wear time and SED (min/day) shown in the present study. Using a ≥10 h ≥4d criteria, wear time must be expected to differ by >2 hours/day between subsequent weeks. Given the strong relationship between wear time and SED (β = 0.709: i.e., 1 minute change in wear time would translate into ~43 sec change in SED time), adjustment for wear time reduced the variability for SED by ~37%. A relatively strong relationship with wear time was also shown for LPA, whereas weaker (although statistically significant) relationships were found for other variables. This clearly indicates that wear time should be considered a crucial variable in data reduction and analysis of accelerometer data, which is consistent with findings of a recent study [33]. Herrmann et al [33] showed patterns across 10 to 14 hours of wear time in a simulation study based on NHANES data which coincide with the present findings. Thus, our findings, along with those of others, shows that SED and PA as determined by accelerometry, should be either reported as percentage values or, alternatively, analyses could be adjusted for wear time, which might be preferred if appropriate. Studies that have investigated the association between SED and cardio-metabolic health vary considerable in their applied operationalization of SED, using absolute values without adjustment for wear time [14, 19], absolute values with adjustment for wear time [15, 17, 20], percentage values [16, 13] or a combination of several approaches [18, 12]. Thus, variation in reliability might be one source to explain disparate findings across studies.

The present estimates of number of days needed to achieve a reliability of ICC = 0.80 for overall PA and MVPA, fall in between of previous estimates. While several studies have found that 2–6 days are required [5, 7, 8, 6, 30], other studies have shown that 12 [4] and 16–23 days are needed [3]. Jerome et al [3] used a wear time criteria of ≥6 h, which might have led to the need for many days of measurement, in line with the trend across the wear time criteria applied in the present study. Levin et al [4] applied a protocol with measurements during an entire year, which may have introduced greater variation (e.g., seasonal effects) than across subsequent days and weeks. Nevertheless, as ICC is based on variance partitioning and depends on the relative difference in variances (inter- vs. intra-individual sources of variance), the coefficient is context-specific. Thus, it change with the range of observations (similar to the correlation coefficient), despite residual variance and absolute measures of reliability being unaffected [26–28]. The current sample was a convenience sample exhibiting a higher activity level (but with approximately similar heterogeneity as evaluated by SDs for the different outcome variables) as compared to population estimates for the corresponding age group [34–36]. Thus, it could be argued that our estimates of ICC might be fairly generalizable to the general adult population. Yet, previous studies have concluded that it does not seem to be any relationship between activity level and reliability [6, 4]. Based on the heteroscedastic patterns of variability found for overall PA level and MVPA (Fig 1), the current study indicates otherwise. Thus, the sample characteristics (exhibiting a high PA level) might be a likely explanation for the lower reliability found for PA compared to other studies. This means that researchers should determine reliability in their specific samples to make appropriate decisions on valid wear criteria in a given study. Yet, it could be hypothesized from the current study that SED are less influenced by sample characteristics than PA, as a homoscedastic pattern was evident for SED as opposed to the heteroscedastic pattern found for PA.

As expected, the findings presented in Table 2 suggest that increased wear time (both hours per day and the total number of days) will lead to improved reliability. However, this pattern was less pronounced when different wear time criteria was applied to the week-by-week analyses. This finding is consistent with the finding by Jerome et al [3], who detected equivalent estimates of reliability for wear times of ≥6 and ≥10 h/day, as well as for 4 and 7 days of monitoring. Based on this finding, Jerome et al concluded that Spearman-Brown estimates should be interpreted with caution and be secondary to actual reliability estimates. We have no other explanation for such disparate findings than suggesting that averaging several days of measurement can lead to relatively stable estimates of weekly SED and PA despite great variability between days, thus, weekly activity might not be properly modeled by the Spearman-Brown formula. Together, these findings suggest that future studies should determine the week-by-week test-retest reliability of accelerometer measurements, and to a lesser extent rely on estimates based on day-by-day measurements.

Nevertheless, our findings suggest that 6–9 days of measurement might be needed to reliably capture SED and PA, given a wear time criteria of 10–12 h/day. This necessitates an increased monitoring length, compared to that of 7 days that is usually applied. Although this might improve validity of study conclusions, the burden for subjects should be kept minimal to maximize response rate and adherence to the monitoring protocol. Feedback from our subjects clearly indicated that 21 days of monitoring was a lengthy measurement. However, 14 days would be appropriate, under the assumption that subjects obtain 4–5 valid days per week. Still, this is a matter of the research question posed, as population-estimates on a group level requires less reliability than individual-level estimates. Also, an increased wear time per day is an option. However, the number of observations left for analyses declined with increased wear time criteria, as shown previously [37]. Thus, the choice of wear time criteria is a trade-off between reliability and power, of which both are of crucial importance to arrive at valid conclusions, as the loss of both will bias conclusions towards the null-hypothesis. As noise in predictor (x-) variables will lead to attenuation of regression coefficients by biasing coefficients towards the null (regression dilution bias), and noise in outcome (y-) variables will increase standard errors and increase the likelihood of performing type II errors [1], unreliable measures will weaken researchers ability to make valid conclusions in PA epidemiology. If PA is modeled as an exposure variable, increased sample size on the expense of reliability will only make the results more precisely wrong [1].

We found significant, although small, differences in activity level and SED between week days and weekend days. Although previous studies have shown somewhat conflicting findings on this point [7, 8, 6], application of a wear day criteria requiring the inclusion of at least one weekend day may be justifiable based on the current study.

### Strengths and limitations

We included a relatively large sample which completed a 21-day monitoring protocol. To the best of our knowledge, we are the first to present absolute measures of week-by-week reliability for SED and PA obtained by accelerometry. Yet, subjects were not a representative draw of the general population, thus, future studies should assess reliability in larger studies with representative samples. As the heterogeneity of the sample is crucial for the ICC obtained [26–28], a representative sample would be superior in terms of interpretation of the reliability coefficients. Yet, absolute measures of reliability (e.g., SEM and LoA) are not influenced by the range of observations.

## Conclusions

We conclude that more than 7 days of measurement was needed to achieve a reliability (ICC) of 0.80 in the current study, depending on the variable(s) of interest. Although we obtained a reliability of ICC >0.70 for 3 subsequent weeks of measurement using frequently applied criteria for wear time, considerable week-by-week variability was found. The findings clearly demonstrated that wear time was of crucial importance to reliably assess SED. Thus, contrary to our hypothesis, the reliability of assessing SED and PA were similar, when wear time was appropriately corrected. Researchers need to be aware of intra-individual variability in accelerometer-measurements and take appropriate actions according to the hypothesis under study, because noise in any measurement will attenuate “real” relationships between PA and health and increase the likelihood of performing type II errors. In terms of the number of monitoring days needed, this is a trade-off between reliability and statistical power.

## Supporting Information

S1 Dataset Supplementary data file including all material underlying the present study. (SAV)
